# Cost effectiveness and affordability of trastuzumab in sub-Saharan Africa for early stage *HER2*-positive breast cancer

**DOI:** 10.1186/s12962-019-0174-7

**Published:** 2019-02-28

**Authors:** Noga Gershon, Yakir Berchenko, Peter S. Hall, Daniel A. Goldstein

**Affiliations:** 1 Ben-Gurion University of the Negev, P.O.B. 653, Beer-Sheva, 8410501 Israel; 2 Edinburgh Cancer Research Centre, Western General Hospital, Edinburgh, UK; 3 Davidoff Cancer Center, Rabin Medical Center, Petach Tikvah, Israel; 4 Department of Health Policy and Management, University of North Carolina, Chapel Hill, USA

**Keywords:** Cost-effectiveness, Trastuzumab, Sub-Saharan Africa

## Abstract

**Background:**

Breast cancer is the second most common cancer worldwide, the most common among women, and the most frequent cause of death among women in less developed regions. Trastuzumab is a humanized monoclonal antibody that downregulates the extracellular domain of the *HER2* protein. Using trastuzumab to treat women with localized *HER2*-positive breast cancer has been shown to improve survival. The objective of this study is to explore the cost-effectiveness of adjuvant trastuzumab, from a societal perspective, in 11 African countries. In addition, we aimed to establish value-based prices for trastuzumab based on the gross domestic product per capita in each country.

**Methods:**

We developed a Markov model in order to assess the costs and benefits associated with trastuzumab treatment over a lifetime horizon. A probabilistic sensitivity analysis was performed in order to estimate the impact of uncertainty of parameter-values on the results. Efficacy inputs were derived using clinical trial data from non-African countries.

**Results:**

In the base case analysis, trastuzumab yielded a gain ranging from 0.92 LYs in Nigeria to 1.07 LYs in South Africa, and 0.9 QALYs in Nigeria to 1.02 QALYs in South Africa. The incremental cost ranged from 19,561 USD in Nigeria to 19,997 USD in Congo, and an incremental cost-effectiveness ratio ranging from 19,534 USD/QALY in South Africa to 21,697 USD/QALY in Nigeria. Using willingness to pay estimates based on World Health Organization recommendations, trastuzumab appear to not be cost-effective in all countries analyzed. Cost-effectiveness estimates were most sensitive to the discount rate, trastuzumab cost, and the hazard ratio.

**Conclusions:**

Trastuzumab does not appear to be cost effective in the African countries analyzed. In order for trastuzumab to be cost-effective, the costs of treatment would require significant discounts.

**Electronic supplementary material:**

The online version of this article (10.1186/s12962-019-0174-7) contains supplementary material, which is available to authorized users.

## Background

Non-communicable diseases, are responsible for about 70% of deaths worldwide. It was predicted that by 2050, 24 million people will be diagnosed with cancer annually. Among them, up to 70% will be from low-income and middle-income (LMICs) countries [1]. The increasing incidence in these countries is due to lifestyle changes, increased life expectancy, and the improvements in treating infectious diseases [1]. Health care systems in many African countries are struggling to deal with the increasing demand caused by the increasing number of cancer patients. Furthermore, using the same care guidelines from high-income countries in a region with less resources and fewer personnel is inappropriate. The high mortality of cancer patients in Africa is multifactorial—due to poor infrastructure, lack of skilled health-care workers, advanced stage at diagnosis, reliance on traditional therapy, few treatment options, and poor compliance [2]. According to a report published by the Institute of Medicine (IOM), the burden of cancer is growing in many poor countries [3]. Breast cancer is the second most common cancer worldwide, and the most common among women. Moreover, breast cancer is the fifth most common cause of death overall, the most frequent cause of death among women in less developed regions, and the second cause of death in more developed regions [4].

Trastuzumab is a humanized monoclonal antibody that downregulates the extracellular domain of the *HER2* protein. Trastuzumab significantly increases the cure rate in patients with *HER2* positive localized breast cancer, and has therefore become a standard adjuvant treatment for early stage breast cancer in many countries [5]. In a survey conducted on breast cancer management in Africa, trastuzumab was available in 10 out of 19 facilities. However, only 5% of the patients were able to afford it [6]. Due to minimal availability of data, there is very scant evidence regarding actual treatment patterns in sub-Saharan Africa. Furthermore, it was found that many breast cancer patients in sub-Saharan Africa are usually treated with tamoxifen regardless of their receptor status [2].

The aim of this study was to explore the cost-effectiveness and affordability of adjuvant trastuzumab treatment for *HER2*-positive breast cancer in 11 countries in sub-Saharan Africa, from a societal perspective. The decision whether an intervention is good value for money or not is determined by comparing the incremental cost-effectiveness ratio result (ICER) in each country to a specific willingness to pay (WTP) threshold [7]. In LMICs, the threshold suggested by the World Health Organization (WHO) is related to the annual gross domestic product (GDP) per capita of each country. If the ICER of the intervention is less than 1xGDP per capita, the intervention is considered as very cost-effective. If the ICER is between 1 and 3× GDP per capita, the intervention is considered cost-effective. Otherwise, it is considered not cost-effective [7]. In order to use WHO’s suggested threshold, the ICER should be in terms of cost per DALY (disability-adjusted life year) [7], however according to a study that reviewed the cost-effectiveness literature, it was found that cost-per quality adjusted life year (QALY) is used to address diseases in wealthier countries such as cancer, whereas cost-per DALY tend to address more prevalent diseases in low income countries such as HIV. Furthermore, QALYs tend to be used for interventions that evaluate pharmaceuticals, while DALYs are used to evaluate interventions that are more focused on immunizations [8]. Additionally, the use of a figure close to the GDP per capita as a cost-effectiveness threshold is widespread in many countries. For example, the United States cost-effectiveness ratio, as for 2016 was 50,000 USD per QALY, while their GDP per capita was 57,588 USD. The United Kingdom cost-effectiveness threshold as for 2016, was set to be 20,000–30,000 GBP/QALY (~ 25,245–37,867 USD per QALY), and the GDP per capita was 40,412 USD [9]. The Netherlands cost-effectiveness threshold was set to 40,000 EUR per QALY (~ 45,498 USD), and the GDP per capita was 45,637 USD [10]. Australia’s GDP per capita is 53,800 USD, however they do not have a specific threshold for funding a new medicine. However, it is more likely that a new drug that costs less than 50,000 USD per QALY will recommended for funding [11]. In Mexico, when deciding whether or not to include a certain technology in the public healthcare system, the GDP per capita is defined to be the cost-effectiveness threshold [12]. This threshold is also being used in Chile [13] and Colombia [14] to define a technology as cost-effective. Following that, we decided to use the GDP per capita threshold to determine whether trastuzumab is cost-effective or not in sub-Saharan Africa. Furthermore, with DALY being typically an intermediate between LY and QALY, since in our paper the LY and QALY obtained are not far apart (and thus, replacing either by DALY will not change much), the extra page-space and demand from the reader seem unwarranted. An additional objective of this paper was to estimate a value-based price (VBP) for each country based on each country’s GDP per capita.

## Methods

We developed a Markov model with monthly cycles and life time horizon using the Rstudio platform. The model was used to estimate the costs and health outcomes (LYs, QALYs) associated with two treatment strategies (chemotherapy with and without trastuzumab) for treating early stage *HER2* positive breast cancer. The chemotherapy regimen used in this model is anthracycline based chemotherapy as depicted in the HERA trial [15, 16]. However, there are multiple different approaches regarding timing and precise therapy such as the use of taxane based therapy, depending on the patient and stage of disease. However, as the precise treatment patterns in sub-Saharan Africa are unknown, it would only be speculation to suggest that one chemotherapy is used in preference of another. This limits the applicability of this model.

### Model structure

The Markov model was developed using the model structure and data used in a recently published study concerning the cost-effectiveness of trastuzumab in South-America [17]. The structure of the model was quite similar to several previously published cost-effectiveness analyses concerning trastuzumab [18–21]. The HERA trial is considered by many to be the most pivotal trial due to the statistically significant results. There have however been considerations that a shorter duration of treatment may be a reasonable option. The FinnHER study evaluated the use of trastuzumab for only 9 weeks compared to no trastuzumab [22]. However this short duration was not compared to the longer duration and did not become accepted as a standard of care around the world. One exception to this was in New Zealand, where the government initially provided 9 weeks of therapy, but then decided to provide 12 months of therapy [23] following demonstration of lack of non-inferiority with the shorter course [24].

Other studies such as the PERSEPHONE trial [25] have assessed the efficacy of using 6 months of trastuzumab, however this has still not become the standard of care due to multiple statistical considerations. A recent meta-analysis suggested preferential clinical outcomes with 12 months of therapy.

**Fig. 1:**
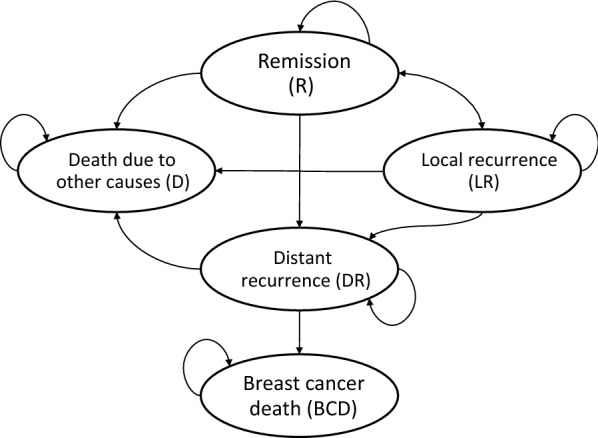
Markov model structure

The inputs of the model were based upon the HERA trial [15, 16, 26] which was used to construct prior cost-effectiveness analyses. Even though the HERA trial was conducted in HIC, we had no reason to believe that the efficacy and side effect profile are different among different ethnic groups. The model illustrated in Fig. 1, consists of five different states: “Remission” (R), “Loco-regional recurrence” (LR), “Distant recurrence” (DR) including metastasis, “Breast cancer death” (BCD), and “Death due to other causes” (D). Patients enter the model from the “Remission” state, they can move to “Loco-regional recurrence” (LR) and return to “Remission” after a successful treatment. They can move from the “Remission” (R) state to “Distant recurrence” (DR) when metastasis is developed. Patients in “Loco-regional recurrence” (LR) state can move to “Distant recurrence” (DR). Patients in all states can move to the “Death due to other causes” (D) state due to all-cause mortality. Only patients in “Distant recurrence” (DR) state can move to “Breast cancer death” (BCD). The influence of trastuzumab on the patients was modeled by changing the transition probabilities from R to DR and from LR to DR for the trastuzumab arm, using the hazard ratio. The probability of moving from DR to BCD is identical for both arms. This is because using trastuzumab only delays or prevents a patient from moving to the DR state. Once a patient has arrived in the DR state, the probability of staying in this state, or moving to other states is identical regardless of the patient’s arm. It was assumed that the effect of trastuzumab lasted 5 years and that there were no cancer recurrences after 20 years of follow-up [17]. Moreover, heart failure incidence that was reported in the trastuzumab trials was not incorporated in the model since it is reversible, and is not associated with increased mortality [27, 28].

The model outputs were costs, life years (LYs), QALYs and ICERs. Costs, LYs, and QALYs were discounted at a yearly rate of 3% as recommended by the WHO [29].

### Patient population

For each treatment strategy, a hypothetical cohort of 10,000 patients was simulated.

Patients who enter the model are 45 years corresponding with the median age of breast cancer patients in Africa [30]. Few studies were conducted concerning the clinicopathological and biological characteristics of breast cancer in sub-Saharan Africa [31]. Information on the incidence of *HER2* positive, as presented in different studies, can be found in Table 1. Approximately 25% of breast cancers are *HER2* positive, and only those patients are eligible to receive trastuzumab. Therefore, the model was developed to evaluate the cost-effectiveness of trastuzumab only in *HER2* positive patients. In this study, we assumed all patients have *HER2* positive early stage breast cancer which was completely resected. The background mortality information of the patients was retrieved from the WHO Global Health Observatory data repository [32]. These values are different for patients from different countries (see Additional file 1: Table S1 and Additional file 2: Figure S1).

**Table 1:** HER2 positive incidence

Country	Incidence	Sources
Congo	Not found	
Ethiopia	23%	[31]
Guinea	Not found	
Kenya	26%	[33]
Namibia	Not found	
Nigeria	26%	[34]
Rwanda	26.3%	[35]
Uganda	22%	[36]
Zambia	Not found	
Zimbabwe	Not found	
South Africa	22.50%	[37]

### Costs

The total costs for the trastuzumab treatment were based on a first dose of 8 mg/kg, followed by sixteen doses of 6 mg/kg for women that weigh 60 kg, based on the average weight of women in the selected countries [38, 39], considering 10% drug wastage. The price of trastuzumab treatment was estimated from extensive negotiations made as has been indicated as a realistic discount based on prior negotiations [40]. Since chemotherapy was used in both arms, we decided to disregard its price as it will add up the same amount in both arms costs and it will eventually cancel each other out in the ICER calculation. The prices for the local and distant recurrence states (LR and DR) were estimated from the prices in Bolivia, as previously published [17], normalized by GDP per capita. We assumed that there is no cost for the “Remission” state in African countries (see Table 2).

**Table 2:** Cost inputs

Country/costs (USD)	Trastuzumab treatment	Local recurrence	Distance recurrence
Congo	20,000	974	1356
Ethiopia	20,000	1323	1841
Guinea	20,000	1136	1581
Kenya	20,000	2769	3853
Namibia	20,000	9719	13,524
Nigeria	20,000	5446	7578
Rwanda	20,000	1456	2027
Uganda	20,000	1424	1981
Zambia	20,000	2695	3751
Zimbabwe	20,000	2090	2909
South Africa	20,000	11,836	16,470

GDP per capita values were retrieved from the World Bank database [41].

### Quality of life

We incorporated quality of life data from previously published data and these are presented in Table 3 [17].

**Table 3:** Model parameters used in the base case analysis and distributions used for the sensitivity analysis

Description	Value	Distribution	Sources
Utilities			
Remission (R)	0.94	Beta (89, 6)	[42, 43]
Local recurrence (LR)	0.82	Beta (77, 23)	[16]
Distant recurrence (DR)	0.58	Beta (171, 79)	[16]
Transition probabilities			
R > LR	0.029	Beta (27, 983)	[26]
R > DR	0.087	Beta (102, 1061)	[15, 16]
LR > R	0.1	Beta (111, 899)	[16]
LR > DR	0.261	Beta (119, 931)	[16]
DR > BCD	0.325	Beta (15, 20)	[16]
R, DR, LR > D	All-cause mortality	Age-specific mortality	[32]
Hazard ratio years 1–5	0.59	Log-Normal (− 0.527, 0.089)	[16, 44]

### Sensitivity analyses

In order to assess the robustness of the model, a probabilistic sensitivity analysis was performed in order to assess the impact of uncertain variables on the results. Each variable was varied separately according to the probability distribution of each variable as described in Table 3.

## Results

In the base case analysis, trastuzumab yielded a gain ranging from 0.92 LYs in Nigeria to 1.07 LYs in South Africa, and 0.9 QALYs in Nigeria to 1.02 QALYs in South Africa. The ICER ranged from 19,534 USD/QALY in South Africa to 21,697 USD/QALY in Nigeria. Results are presented in Table 4 and in Additional file 3: Figure S2.

**Table 4:** Mean base case results

Country	Results	LYs	QALYs	Costs (USD)	ICER ($/QALY)	ICER ($/LY)
Congo	No Tzb arm	8.88	7.72	2588	20,520	19,701
	Tzb arm	9.89	8.69	22,585		
	Diffrence	1.01	0.97	19,997		
Ethiopia	No Tzb arm	8.87	7.71	3541	19,990	19,384
	Tzb arm	9.90	8.70	23,457		
	Diffrence	1.03	1.00	19,916		
Guinea	No Tzb arm	8.65	7.52	2955	20,692	20,025
	Tzb arm	9.65	8.48	22,898		
	Diffrence	1.00	0.96	19,943		
Kenya	No Tzb arm	8.96	7.79	7467	19,601	18,709
	Tzb arm	10.03	8.80	27,448		
	Diffrence	1.07	1.02	19,982		
Namibia	No Tzb arm	8.69	7.54	25,549	19,818	19,125
	Tzb arm	9.72	8.53	45,173		
	Diffrence	1.03	0.99	19,624		
Nigeria	No Tzb arm	8.51	7.39	14,066	21,697	21,321
	Tzb arm	9.43	8.29	33,628		
	Diffrence	0.92	0.90	19,561		
Rwanda	No Tzb arm	8.98	7.80	3919	19,751	19,004
	Tzb arm	10.03	8.81	23,894		
	Diffrence	1.05	1.01	19,975		
Uganda	No Tzb arm	8.79	7.63	3793	20,477	19,806
	Tzb arm	9.80	8.61	23,738		
	Diffrence	1.01	0.97	19,945		
Zambia	No Tzb arm	8.75	7.60	7116	20,330	19,473
	Tzb arm	9.77	8.58	27,074		
	Diffrence	1.02	0.98	19,958		
Zimbabwe	No Tzb arm	8.57	7.44	5458	20,537	20,086
	Tzb arm	9.56	8.41	25,303		
	Diffrence	0.99	0.97	19,845		
South Africa	No Tzb arm	8.76	7.61	31,160	19,534	18,719
	Tzb arm	9.83	8.63	51,119		
	Diffrence	1.07	1.02	19,960		

The standard error for LYs, QALYs, trastuzumab cost and no trastuzumab cost ranged from 0.052 LYs to 0.056 LYs, 0.049 QALYs to 0.053 QALYs, 27 USD to 328 USD, and 27 USD to 324 USD respectively

**Fig. 2:**
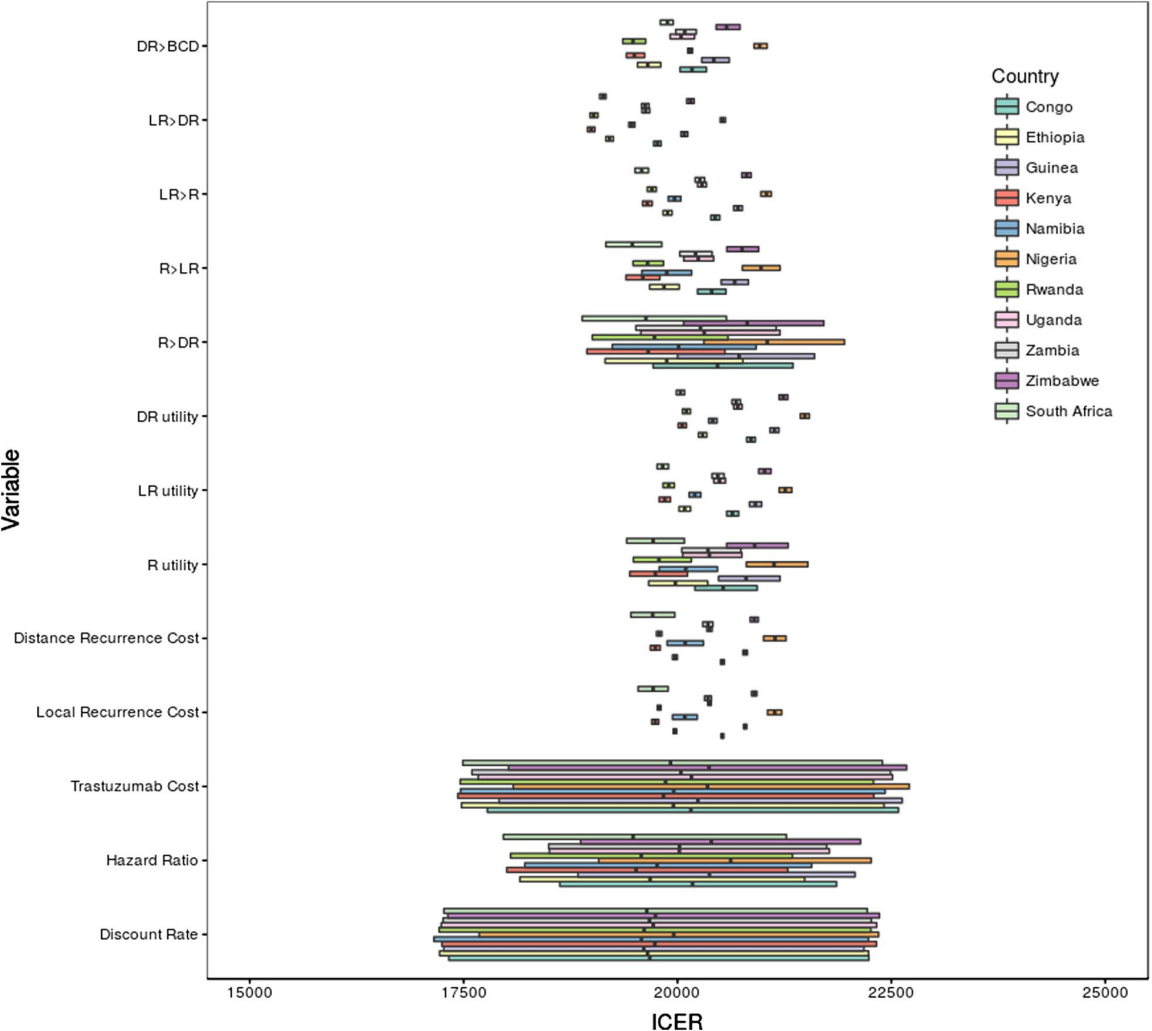
Sensitivity analysis results

Uncertain values of the model were varied in a one-way probabilistic sensitivity analysis. Results for all countries are presented in Fig. 2. The diagram presents ICER results from the 0.25 percentile to the 0.75 percentile. The probabilistic sensitivity analysis results were consistent through all 11 countries. The most sensitive variables were the discount rate, trastuzumab cost, and the hazard ratio. Furthermore, as can be seen in the figure, trastuzumab is not considered cost-effective even with the low ICER values in the sensitivity analysis. Even though the prices used through most countries are identical as well as the drug efficacy, the ICER of each country is different due to the variation in background mortality of each country.

**Fig. 3:**
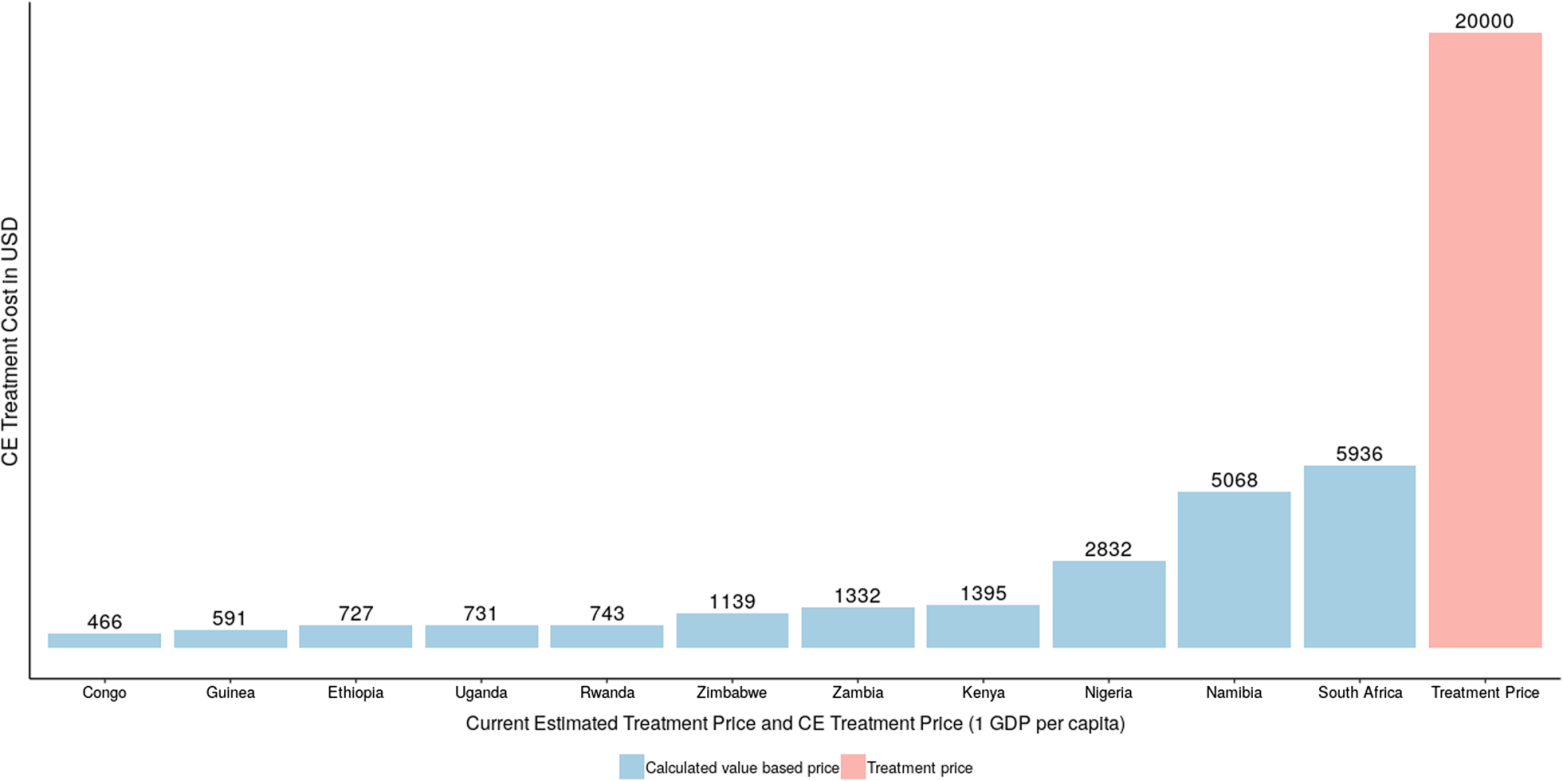
Estimated treatment price in all examined countries compatible with value-based price (1 GDP per capita)

**Fig. 4:**
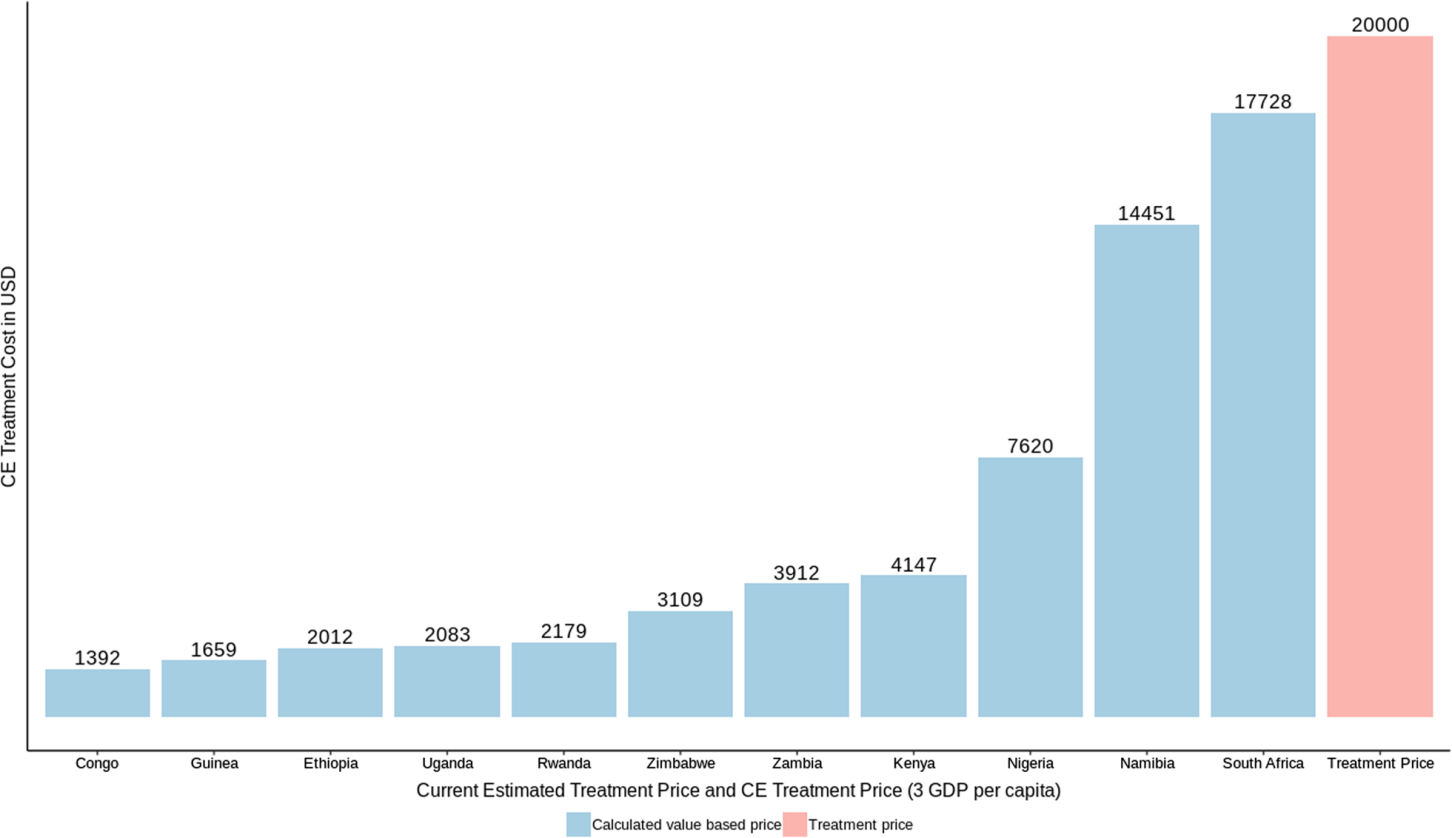
Estimated treatment price in all examined countries compatible with value-based price (3 GDP per capita)

The individual estimates of valued based prices for trastuzumab are presented in Figs. 3, 4 and in Table 5. Since it was concluded that trastuzumab is not cost-effective in the examined countries, we calculated the VBP in such a way that would make the treatment considered as cost-effective in each country, when the WTP threshold is one GDP per capita. In order for the treatment to be considered as cost-effective in African countries, significant price reductions would be required.

**Table 5:** Value based price for each country

Country	Value based price (1 GDP per capita)	Value based price (3 GDP per capita)
Congo	466	1392
Ethiopia	727	2012
Guinea	591	1659
Kenya	1395	4147
Namibia	5068	14,451
Nigeria	2832	7620
Rwanda	743	2179
Uganda	731	2083
Zambia	1332	3912
Zimbabwe	1139	3109
South Africa	5936	17,728

## Discussion

In this paper, the cost-effectiveness of trastuzumab for treating breast cancer was examined. Trastuzumab was compared to conventional chemotherapy treatment. In this analysis, trastuzumab treatment yields a gain ranging from 0.92 LYs in Nigeria to 1.07 LYs in South Africa, and 0.9 QALYs in Nigeria to 1.02 QALYs in South Africa, with an incremental cost ranging from 19,561 USD in Nigeria to 19,997 USD in Congo, and an incremental cost-effectiveness ratio ranging from 19,534 USD/QALY in South Africa to 21,697 USD/QALY in Nigeria per gained QALY.

The ICER results were comparable to previously published results [19, 43–45]. Prior analyses in the UK, Australia, United States and Italy yielded similar results to those in our analysis. Other papers presented higher ICERs, which may be due to calculated costs and survival rates in Africa being significantly lower [18, 46, 47].

Although the ICER results are quite similar to other studies, their meaning is different when examined in Africa. While trastuzumab can be considered as cost-effective in HICs [5, 21, 42–44, 48], this is not the case in LMICs. ICER results in HICs were close or below the suggested threshold by WHO, one GDP per capita, as can be seen in Table 6, while the calculated ICERs in Africa were significantly higher than one GDP per capita for each examined country.

**Table 6:** ICER results and their corresponding GDP per capita

Country	ICER	GDP per capita (USD)
Switzerland	40,505 EUR (45,950 USD) per LYG	57,579
United Kingdom	25,803 GBP (33,073 USD) per QALY	44,252
Sweden	41,500 USD per QALY	46,256
Norway	35,974 EUR (40,810 USD) per LYG	85,170
United States	26,417 USD per QALY	48,061
United States	39,892 USD per QALY	55,443

This study has several limitations. Since no clinical trials were performed in Africa, there was a lack of information regarding the efficacy of the drug in the population in this area. Therefore, for this analysis it was assumed that the findings from the performed clinical trials are relevant in Africa as well. Additionally, although the median age of patients in the HERA trial was 49, we used a different age (45) in our model under the assumption that the reported hazard ratio and the transition probabilities depend on disease progression rather than the age. Furthermore, as indicated by previous literature [30], 45 years old is more representative of the general population of patients in sub-Saharan Africa. Another limitation concerns the omission of heart failure from the model. Although heart failure is reversible and is not associated with increased mortality it may have a mild effect on quality of life estimates. However, since the incidence is very low [27, 28], we excluded it as it would have only a very minor impact on the results of the model. Moreover, given that the gap between the current results and the cost-effectiveness threshold is very large, it can be assumed that the inclusion of heart failure will not change the results significantly. Moreover, as stated before, the transition probabilities were based on the HERA trial, which was conducted in HICs. These probabilities were used due to lack of trials performed in sub-Saharan Africa. Therefore, it can be assumed that in reality these probabilities would be a bit different due to differences between health care systems in HICs and sub-Saharan Africa. Furthermore, not all the countries in Africa provide trastuzumab due to its high price. There is a significant lack of price transparency globally. Hence, the prices for all examined countries were merely estimated. Lastly, since we concluded trastuzumab is not cost-effective in the current setting (12 months treatment), further research concerning a recently suggested treatment duration (6/9 months treatment/9 weeks) [24, 49] needs to be further investigated and maybe adopted in sub-Saharan Africa.

Africa is facing a major public health challenge from non-communicable diseases. Even though infectious diseases are still a major concern in Africa, the overall disease burden proportion in Africa associated with cancer is rising. It was predicted that by 2030, this region will experience an increase of more than 85% cancer burden [50]. One way to improve the current state in Africa is to make cancer drugs more accessible and affordable. Major improvements in the health system and cancer awareness are also crucial.

## Conclusions

In this work we calculate the expected ICER gained by using trastuzumab in sub-Sahara Africa, and compare it to the threshold recommended by the WHO. Although the ICER results are quite similar to other studies, their meaning is different when examined in sub-Sahara Africa. While a trastuzumab can be considered as cost-effective in HICs, this is not the case in LMICs. ICER results in HICs were close or below the suggested threshold by WHO, one GDP per capita, while the calculated ICERs in Africa were significantly higher than one GDP per capita for each examined country.

Despite several limitations of our study, it seems that a significant reduction in trastuzumab’s price (compared to its price in HICs) would be required in order for it to be cost-effective in sub-Sahara Africa.

## Additional files

**Additional file 1: Table S1.** Probability of dying between ages x and x + 5 for each country.

**Additional file 2: Figure S1.** Probability of dying between ages x and x + 5 for each country.

**Additional file 3: Figure S2.** ICER (incremental cost-effectiveness ratio) results for each country.

## Abbreviations

QALY: quality-adjusted life year
LY: life-year
LMICs: low-income and middle-income
ICER: incremental cost-effectiveness ratio result
Tzb: trastuzumab
WTP: willingness to pay
WHO: World Health Organization
GDP: gross domestic product
DALY: disability-adjusted life year
VBP: value-based price
