# Supplementary material for: Cost effectiveness and affordability of trastuzumab in sub-Saharan Africa for early stage HER2-positive breast cancer
Source: Cost Eff Resour Alloc. 2019 Feb 28;17:5. doi: 10.1186/s12962-019-0174-7 (PMC6396469; doi:10.1186/s12962-019-0174-7)
Supplement: Supplementary file 1 — Additional file 1: Table S1. Probability of dying between ages x and x + 5 for each country. [file 12962_2019_174_MOESM1_ESM.docx]

**Additional file 1: Table S1.** Probability of dying between ages x and x + 5 for each country.

| Age Group | Congo | Ethiopia | Guinea | Kenya | Namibia | Nigeria | Rwanda | Uganda | Zambia | Zimbabwe | South Africa |
| --- | --- | --- | --- | --- | --- | --- | --- | --- | --- | --- | --- |
| 45-49 years | 0.042 | 0.03 | 0.042 | 0.03 | 0.044 | 0.049 | 0.027 | 0.04 | 0.041 | 0.055 | 0.037 |
| 50-54 years | 0.051 | 0.039 | 0.053 | 0.039 | 0.051 | 0.061 | 0.036 | 0.05 | 0.049 | 0.062 | 0.046 |
| 55-59 years | 0.061 | 0.051 | 0.068 | 0.05 | 0.063 | 0.08 | 0.047 | 0.061 | 0.06 | 0.068 | 0.063 |
| 60-64 years | 0.087 | 0.078 | 0.099 | 0.076 | 0.088 | 0.137 | 0.071 | 0.087 | 0.086 | 0.09 | 0.09 |
| 65-69 years | 0.133 | 0.124 | 0.151 | 0.118 | 0.133 | 0.211 | 0.113 | 0.134 | 0.131 | 0.133 | 0.132 |
| 70-74 years | 0.208 | 0.197 | 0.232 | 0.183 | 0.211 | 0.33 | 0.183 | 0.209 | 0.203 | 0.203 | 0.192 |
| 75-79 years | 0.316 | 0.304 | 0.343 | 0.271 | 0.328 | 0.486 | 0.286 | 0.318 | 0.309 | 0.308 | 0.272 |
| 80-84 years | 0.465 | 0.453 | 0.492 | 0.386 | 0.49 | 0.666 | 0.436 | 0.465 | 0.458 | 0.457 | 0.404 |
| 85+ years | 1 | 1 | 1 | 1 | 1 | 1 | 1 | 1 | 1 | 1 | 1 |

## The data was retrieved from the WHO Global Health Observatory data repository [32].
