# Supplementary figures and images for: Cost effectiveness and affordability of trastuzumab in sub-Saharan Africa for early stage HER2-positive breast cancer
Source: Cost Eff Resour Alloc. 2019 Feb 28;17:5. doi: 10.1186/s12962-019-0174-7 (PMC6396469; doi:10.1186/s12962-019-0174-7)

**Additional file 2: Figure S1.** Probability of dying between ages x and x + 5 for each country.


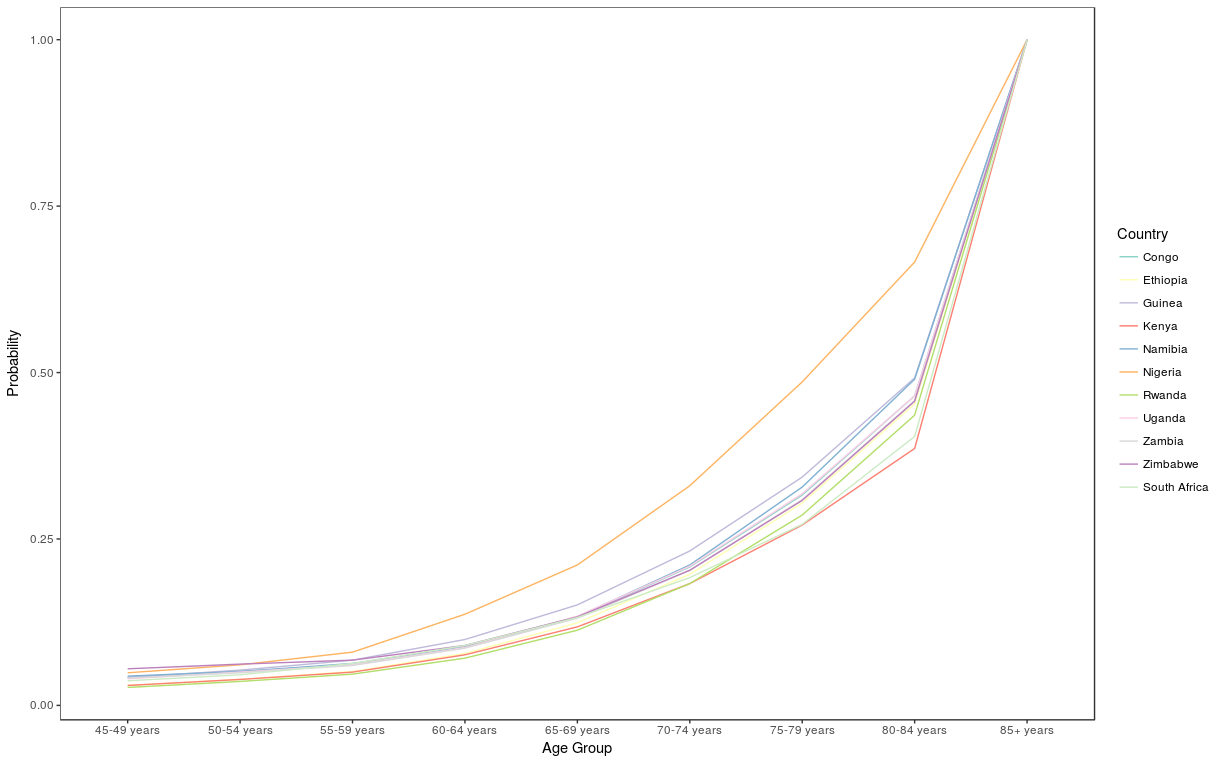

Supplement: Supplementary file 2 — Additional file 2: Figure S1. Probability of dying between ages x and x + 5 for each country. [file 12962_2019_174_MOESM2_ESM.docx]

**Additional file 3: Figure S2.** ICER (incremental cost-effectiveness ratio) results for each country.

*
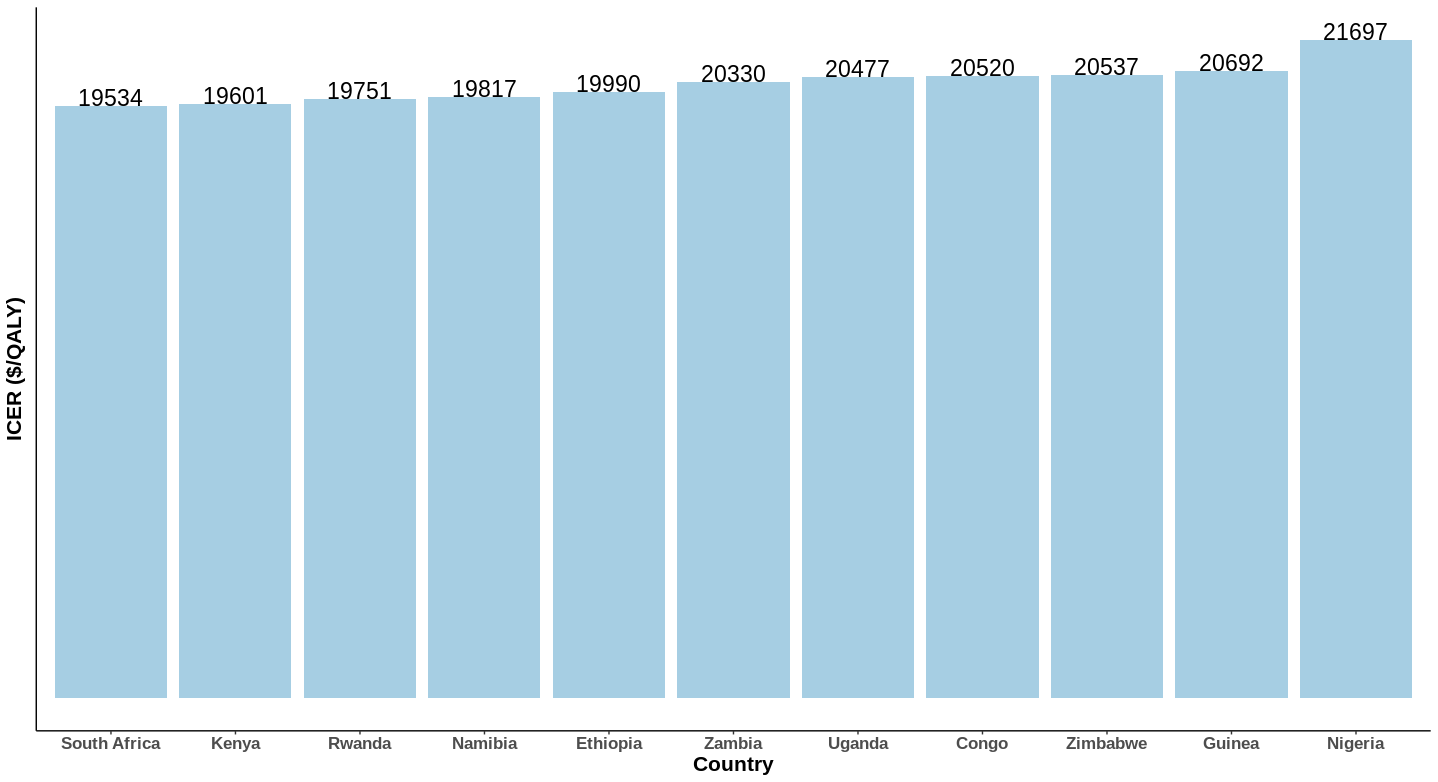
*

Supplement: Supplementary file 3 — Additional file 3: Figure S2. ICER (incremental cost-effectiveness ratio) results for each country. [file 12962_2019_174_MOESM3_ESM.docx]
